# Simulation of electricity consumption data using multiple artificial intelligence models and cross validation techniques

**DOI:** 10.1016/j.dib.2023.109718

**Published:** 2023-10-24

**Authors:** Mariam Hosny, Omnia Abu Waraga, Manar Abu Talib, Mohamed Abdallah

**Affiliations:** a Department of Civil and Environmental Engineering, University of Sharjah, United Arab Emirates; b Department of Computer Science, University of Sharjah, United Arab Emirates

**Keywords:** Electricity consumption prediction, Machine learning models, Artificial neural network, Cross validation

## Abstract

Worldwide, electricity production exceeds its consumption which leads to wasted financial and energy resources. Machine learning models can be utilized to predict the future consumption to avoid these significant losses. This paper presents the data for the monthly electricity consumption on the community level during May 2017–December 2019 in Dubai, United Arab Emirates. It was acquired from Dubai Pulse, an online repository containing consumption data from Dubai Electricity and Water Authority which provides utility services to the Emirate. Multiple parameters, such as population and number of buildings, were acquired from Dubai Statistics Center in addition to temperature which was obtained from Dubai International Airport. Additional features, such as expatriate ratio, number of customers, and building occupancy, were computed from the available data and utilized to generate a dataset towards accurate prediction. Various linear regression variants, support vector machines, decision tree models, ensemble models, and neural networks were implemented to forecast electricity consumption. The models were trained on two different formats of the same dataset, which were generated by sorting the data with respect to time, named as temporally ordered dataset, and by randomly dividing the data, labelled as randomly split dataset. In addition, the dependence of the models on the amount of data was identified by varying the size of the testing data. Moreover, two cross-validation (CV) procedures, namely rolling CV method and moving CV method, were applied to assess the reliability of the models. All analyses were evaluated by utilizing several performance metrics, namely root mean squared error, coefficient of determination, i.e., R^2^, 10-fold CV score, mean absolute error, median absolute error, and computational time. Furthermore, this data could be utilized to analyze the effect of coronavirus disease 2019 (COVID-19) prevention measures in Dubai on electricity usage as well as evaluate the consumption patterns at the consumer level.

Specifications Table

**Table:** 

Subject	Energy
Specific subject area	Power Consumption, Forecasting Methods, Applied Machine Learning, Big Data Analytics.
Data format	Raw, Filtered, Analysed
Type of data	Table, Figure
Data collection	Monthly electricity consumption and temperature records as well as annual statistics for population and buildings were acquired. Number of sites were computed as number of accounts with consumption greater than zero. Expatriate ratio was calculated as the ratio of expatriate residential sites to all residential sites. Building occupancy was calculated as population divided by number of buildings.
Data source location	Electricity consumption and Shams Dubai consumption ­ Institution: Dubai Electricity and Water Authority ­ City: Dubai ­ Country: United Arab Emirates Population and number of buildings ­ Institution: Dubai Statistics Center ­ City: Dubai ­ Country: United Arab Emirates Temperature ­ Institution: Dubai International Airport ­ City: Dubai ­ Country: United Arab Emirates
Data accessibility	Primary data sources: ­ Electricity consumption and Shams Dubai consumption records from Dubai Pulse online repository [ref: https://www.dubaipulse.gov.ae/organization/dewa] ­ Population and number of buildings from Dubai Statistics Center Platform [ref: https://www.dsc.gov.ae/en-us] ­ Temperature records measures at Dubai International Airport from public archives [ref: 10.17632/pmrwbghxys.1]
	Secondary data source: ­ Repository name: Mendeley Data ­ DOI: 10.17632/4b8vjvc7yw.2 Direct URL to data: https://data.mendeley.com/datasets/4b8vjvc7yw
Related research article	M. Abdallah, M. Abu Talib, M. Hosny, O. Abu Waraga, Q. Nasir, M.A. Arshad, Forecasting Highly Fluctuating Electricity Load using Machine Learning Models Based on Multimillion Observations, Advanced Engineering Informatics, 53 (2022), 101707.

## 1 Value of the Data

• This data is significantly detailed with more than 50 million observations providing the monthly electricity consumption per customer. In addition, essential clarifications are presented in this paper about the consumption data and its behavior.
• Utility service providers can use the data to plan forward for the required energy supply, reduce losses in the grid, and improve the overall efficiency towards energy savings and sustainability. They can achieve this by identifying the communities with fluctuated consumption to prepare plans for load balancing as well as integrating the varying consumption patterns with the different end users, i.e., rate category, to create and implement strict regulations towards reduced consumption.
• The present study analyzes the effect of external factors, such as ambient temperature, annual population and number of buildings per community, number of sites, expatriate ratio, as well as building occupancy, on electricity consumption patterns in fast-growing cities and determines the best-performing machine learning model with the most stability and reliability.
• The historical records of electricity consumption presented in this paper can benefit the scientific community to have robust models that generate accurate forecasts for a specific city and other cities with similar demographic features.
• The data can be further analyzed at the household level to gain insight into customer behavior and consumption patterns by extracting the observations and aggregating them with respect to each household, e.g., contract account.
• This data can be used to train various machine learning models to estimate electricity consumption, such as linear regression variants, support vector machines, decision tree models, ensemble models, and neural networks.

## 2 Data Description

The dataset details the monthly electricity consumption at the community level in Dubai, United Arab Emirates, from May 2017 to December 2019. It consists of electricity consumption records, socio-demographic, and climatic factors obtained from three repositories. The first repository is Dubai Pulse in which Dubai Electricity and Water Authority (DEWA), the electricity provider in Dubai, uploads monthly electricity consumption records for each household account [3,4]. A list of the number of observations obtained from each file is shown in Table 1. As the study aims to evaluate the consumption patterns in Dubai, accounts enrolled in the renewable energy generation program using solar panels, i.e., Shams Dubai, were omitted. This was due to the lack of any numerical relation between electricity consumption and Shams Dubai consumption.

**Table 1:** Description of the number of observations for each data file.

File name	Number of observations
Elec_Data/Electricity_Consumption_2017-05-31_00-00-00.csv	1535,727
Elec_Data/Electricity_Consumption_2017-06-30_00-00-00.csv	1468,448
Elec_Data/Electricity_Consumption_2017-07-31_00-00-00.csv	1620,428
Elec_Data/Electricity_Consumption_2017-08-31_00-00-00.csv	1557,710
Elec_Data/Electricity_Consumption_2017-09-30_00-00-00.csv	1406,342
Elec_Data/Electricity_Consumption_2017-10-31_00-00-00.csv	1579,135
Elec_Data/Electricity_Consumption_2017-11-30_00-00-00.csv	1579,774
Elec_Data/Electricity_Consumption_2017-12-31_00-00-00.csv	1584,323
Elec_Data/Electricity_Consumption_2018-01-31_00-00-00.csv	1595,127
Elec_Data/Electricity_Consumption_2018-02-28_00-00-00.csv	1606,328
Elec_Data/Electricity_Consumption_2018-03-31_00-00-00.csv	1619,606
Elec_Data/Electricity_Consumption_2018-04-30_00-00-00.csv	1621,644
Elec_Data/Electricity_Consumption_2018-05-31_00-00-00.csv	1620,188
Elec_Data/Electricity_Consumption_2018-06-30_00-00-00.csv	1572,764
Elec_Data/Electricity_Consumption_2018-07-31_00-00-00.csv	1541,094
Elec_Data/Electricity_Consumption_2018-08-31_00-00-00.csv	1555,447
Elec_Data/Electricity_Consumption_2018-09-30_00-00-00.csv	1665,602
Elec_Data/Electricity_Consumption_2018-10-31_00-00-00.csv	1673,212
Elec_Data/Electricity_Consumption_2018-11-30_00-00-00.csv	1676,436
Elec_Data/Electricity_Consumption_2018-12-31_00-00-00.csv	1633,389
Elec_Data/Electricity_Consumption_2019-01-31_00-00-00.csv	1689,555
Elec_Data/Electricity_Consumption_2019-02-28_00-00-00.csv	1684,115
Elec_Data/Electricity_Consumption_2019-03-31_00-00-00.csv	1652,701
Elec_Data/Electricity_Consumption_2019-04-30_00-00-00.csv	1729,879
Elec_Data/Electricity_Consumption_2019-05-31_00-00-00.csv	1732,610
Elec_Data/Electricity_Consumption_2019-06-30_00-00-00.csv	1582,286
Elec_Data/Electricity_Consumption_2019-07-31_00-00-00.csv	1762,959
Elec_Data/Electricity_Consumption_2019-08-31_00-00-00.csv	1774,051
Elec_Data/Electricity_Consumption_2019-09-30_00-00-00.csv	1712,123
Elec_Data/Electricity_Consumption_2019-10-31_00-00-00.csv	1750,836
Elec_Data/Electricity_Consumption_2019-11-30_00-00-00.csv	1704,034
Elec_Data/Electricity_Consumption_2019-12-30_00-00-00.csv	1733,034
Elec_Data/Electricity_Consumption_2020-01-31_00-00-00.csv	1826,063
Elec_Data/Electricity_Consumption_2020-02-29_00-00-00.csv	1839,815

**Total Number of Observations**	**55,886,785**

Table 2 lists the attributes of both data sources. Moreover, DEWA has nine rate categories based on the site type, e.g., commercial, industrial, expatriate residential, etc.

**Table 2:** Description of the attributes present in DEWA electricity consumption and shams Dubai files.

Attribute name	Type	Description
Billing Portion	Alpha numeric	A 3-alphanumeric code of the portion that the site belongs to and is used for meter reading purposes
Community	Numeric	A 3-digit code of the community assigned by Dubai Municipality
Rate Category	Text	The type of site, e.g., residential, commercial, industrial, etc.
Consumption Period	Alpha numeric	The date at which the invoice is issued
Calendar Month	Numeric	The month and year in which the electricity was consumed
Contract Account	Numeric	A 9–10 unique identification number of the site
Business Partner	Numeric	An 8–9 identification number for the owner of the site and is common for all the registered sites by the customer
Consumption Unit*	Numeric	Electricity consumption in kWh
Import Unit⁎⁎	Numeric	Electricity imported from the grid in kWh
Export Unit⁎⁎	Numeric	Electricity exported to the grid in kWh

⁎ Not present in DEWA Shams Dubai files.

⁎⁎ Not present in DEWA Electricity Consumption files.

**Fig. 1:**
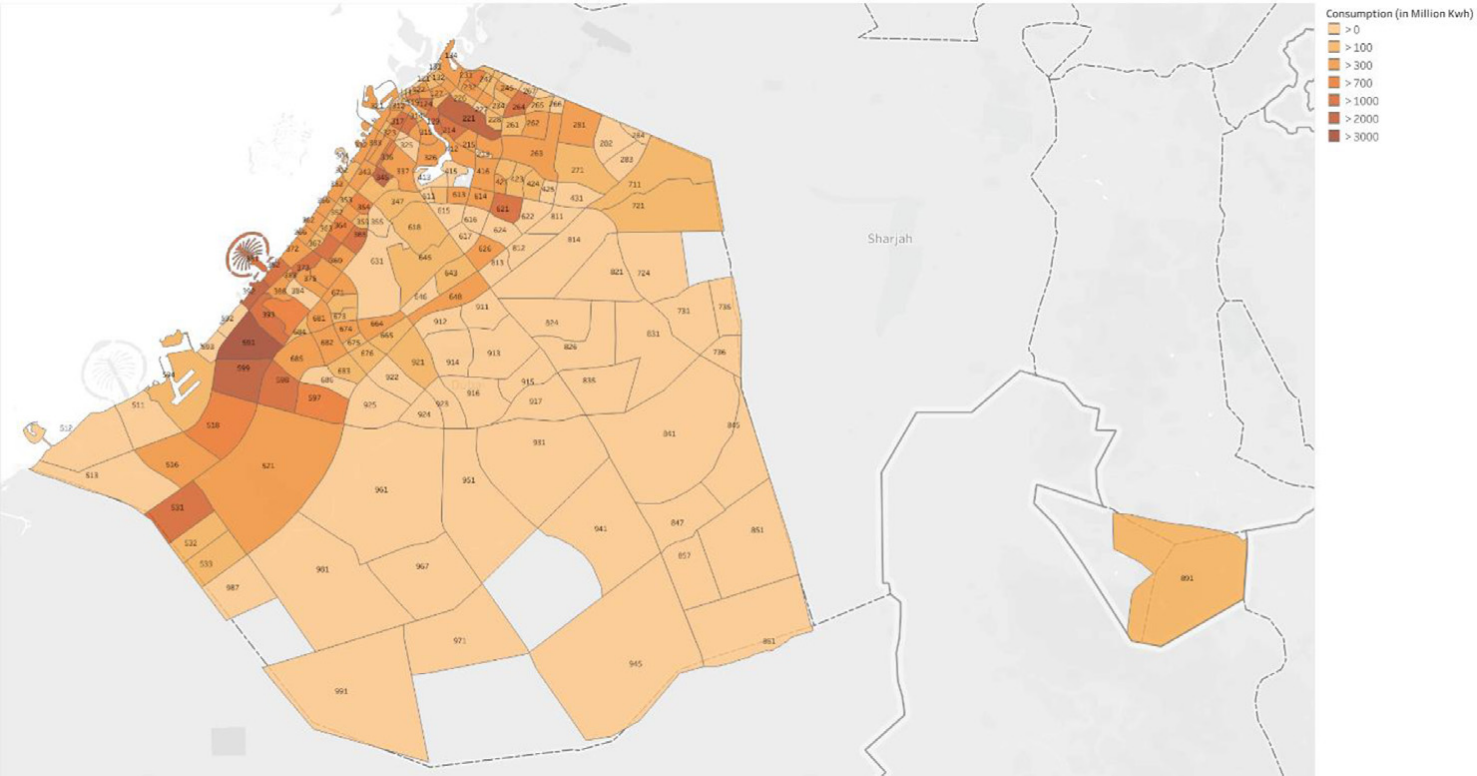
Heat map of the electricity consumption in all communities.

Table 3 describes the present rate categories, as well as the annual number of accounts and energy consumption for each rate category. Fig. 1 presents a heat map for the aggregated electricity consumption of all consumers per community.

**Table 3:** Annual number of accounts and electricity consumption for each rate category.

Rate category	Description	No. of accounts (2017)*	Electricity consumed⁎⁎ (2017)	No. of accounts (2018)	Electricity consumed⁎⁎ (2018)	No. of accounts (2019)	Electricity consumed⁎⁎ (2019)
COMMELEC	Commercial sites	202,951	11,597	223,147	15,052	248,230	15,298
FREENRESIE	Sites with no consumption	197	17	560	87	1017	141
GOVTELEC	DEWA sites	4353	1625	4392	1989	5330	1736
INDTELEC	DEWA residential sites	2519	1567	2637	2186	3883	1924
RESIEXPE	Governmental sites	641,302	6104	764,647	7808	897,550	7983
RESINATE	Industrial sites	38,064	2005	38,956	2470	50,918	2544
RESINATSE	Expatriate residential sites	3027	157	3147	193	3555	199
ENORATE	National residential sites	21	0	16	0	8	0
FREERESIE	National social residential sites	367	33	367	0	2	0

**Total**		892,801	23,105	1037,869	29,785	1210,493	29,825

⁎ From May 2017 to December 2017.

⁎⁎ Electricity measured in million Kilowatt hour (kWh).

**Fig. 2:**
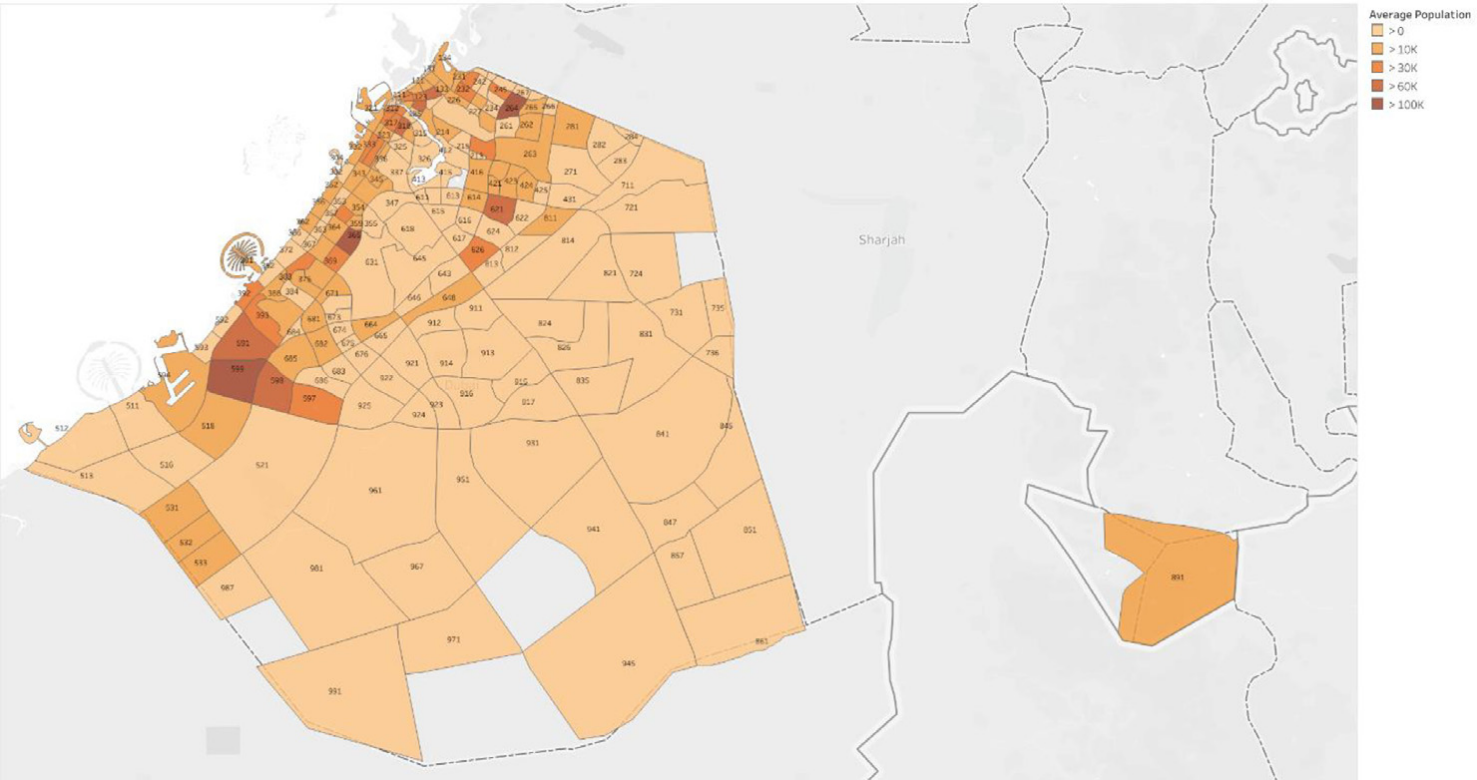
Heat map of the population in all communities.

The second repository is Dubai International Airport where the monthly values of the ambient temperature were acquired for the entire Emirate during the study period [5]. Moreover, the annual population and the number of buildings per community were obtained from the third repository which is Dubai Statistics Center which provides various statistical data from 24 different governmental sectors in Dubai, including demographic, economic, and climatic records [6]. Fig. 2 shows a heat map for the population per community.

**Fig. 3:**
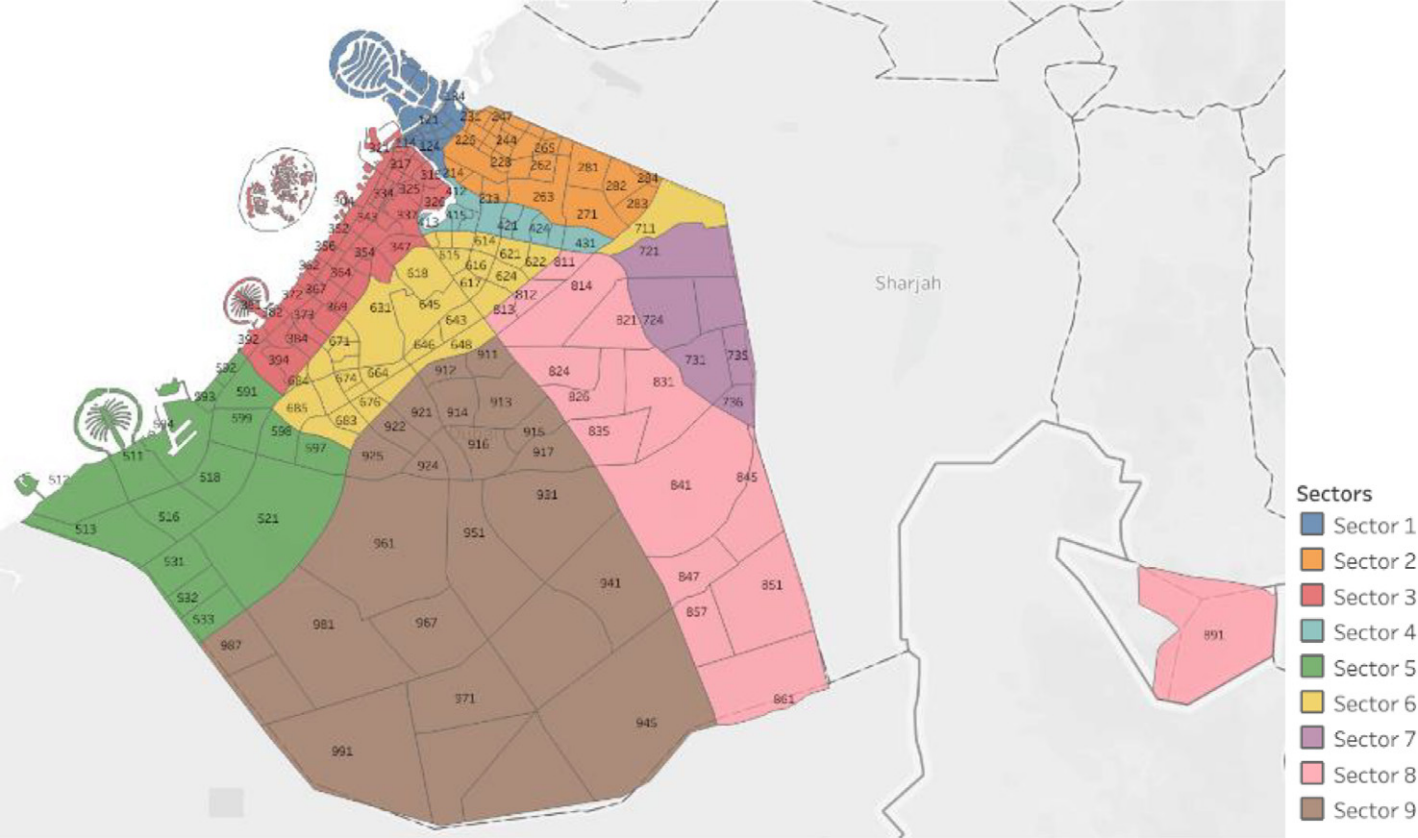
Satellite image for the sectoral distribution of all communities.

Table 4 presents a list of the total number of buildings per building type for the entire city. Overall, Dubai encompasses more than 250 communities, which were divided into nine sectors in this study, as shown in Fig. 3 to facilitate the presentation of the accumulated annual consumption and population in Table 5.

**Table 4:** Summary of the total number of each type of building in 2019.

Type of building	Total	Percentage
One Storey	2199	1.4
Multi-Storey	14,123	8.8
Multi-Storey Ratio	121	0.1
Private Villa	42,743	26.7
Investment Villa	77,802	48.6
Arabic House	5979	3.7
Public	10,405	6.5
Industrial	1055	0.7
Other	5701	3.6
**Total**	160,128

**Table 5:** Summary for the annual sum and average of the population and electricity consumption per sector.

Sector number	Number of communities	Total population⁎⁎			Mean population⁎⁎	Total consumption⁎⁎⁎			Mean consumption⁎⁎⁎
		2017*	2018	2019		2017*	2018	2019	
1	22	4024	5527	5663	5071	2924	3316	3271	3170
2	33	4963	6835	7159	6319	3799	4407	4247	4151
3	56	9722	14,101	14,975	12,933	10,579	12,775	12,901	12,085
4	10	603	928	1001	844	623	753	779	718
5	16	3774	5088	5160	4674	3669	4453	4358	4160
6	32	2677	4384	4770	3944	1984	3029	3266	2760
7	5	77	108	117	101	89	106	99	98
8	16	314	438	468	407	100	191	207	166
9	22	60	84	114	86	83	160	203	149

⁎ From May 2017 to December 2017.

⁎⁎ Population measured in thousands.

⁎⁎⁎ Electricity consumption measured in million kWh.

**Fig. 4:**
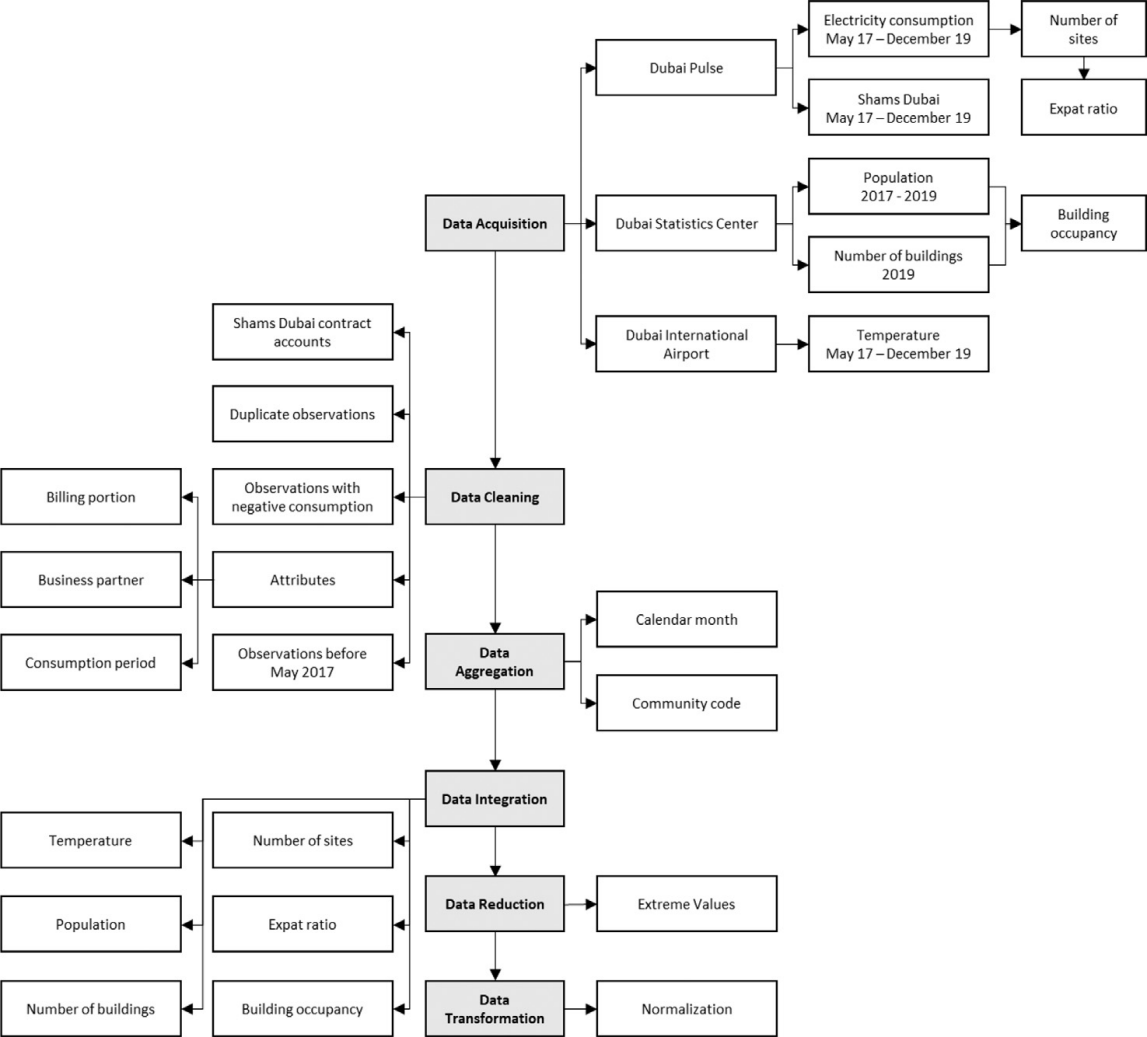
Schematic diagram of the pre-processing methodology.

Additional features were extracted and computed using the acquired data. Pre-processing methods were carried out to aggregate electricity consumption with respect to time and community. Afterwards, the obtained features were added to the dataset, such as temperature, population, number of buildings, number of sites, expatriate ratio, and building occupancy. Next, the necessary data cleaning, integration, and transformation processes were applied in which the lower and upper one percentile were removed, and the electricity consumption values were normalized. Fig. 4 shows the flowchart for the methodology followed to obtain the dataset.

The dataset presented in this article is included in two versions. The first one is “Aggregated Dataset.csv” which consists of the aggregated monthly electricity consumption records on the community level and the exogenous parameters. This dataset is generated using the simulation software [2]. The other is “Final Dataset – Processed.csv” which contains the final dataset generated after applying multiple pre-processing and feature importance steps. This dataset is used to model the electricity consumption predictors presented in the related manuscript [1]. Table 6 shows a brief description and a statistical summary of the mean and range values of all the features in the aggregated dataset after pre-processing. Moreover, the repository includes a supportive file called “Aggregated Electricity and Temperature on City Level.csv” which consist of the aggregated electricity records on the level of Dubai city.

**Table 6:** Descriptive statistical summary of all utilized features in the aggregated cleaned dataset.

Categories	Features	Extracted/Computed	Description	Mean	Range
Electricity	Actual consumption*	Extracted	Actual aggregated electricity consumption⁎⁎	13.23	0.02–89.71
	Normalized consumption	Computed	Normalized actual consumption⁎⁎	0.15	0–1

Socio-demographic	Population	Extracted	Number of residents⁎⁎⁎	17,461	0–202,918
	Number of buildings	Extracted	Number of buildings⁎⁎⁎	865	1–9468
	Number of sites	Computed	Number of sites with a positive consumption⁎⁎	5541	1–37,672
	Building occupancy	Computed	Population divided by total number of buildings⁎⁎⁎	37	0–342
	Expatriate ratio	Computed	Number of expatriate sites divided by number of residential sites⁎⁎	0.72	0–1

Climate	Temperature	Extracted	Actual monthly temperature in Dubai	30.8	20.4–39.2

Spatial	Community code	Extracted	A 3-digit code of the community	–	–

Temporal	Discrete month	Extracted	A value of the month	–	1–12
	Year	Extracted	A value of the year	–	2017–2019
	Continuous month	Computed	A value of the month based on cosine wave	–	–

⁎ Actual electricity consumption measured in million kWh.

⁎⁎ Monthly values per community.

⁎⁎⁎ Annual values per community.

**Fig. 5:**
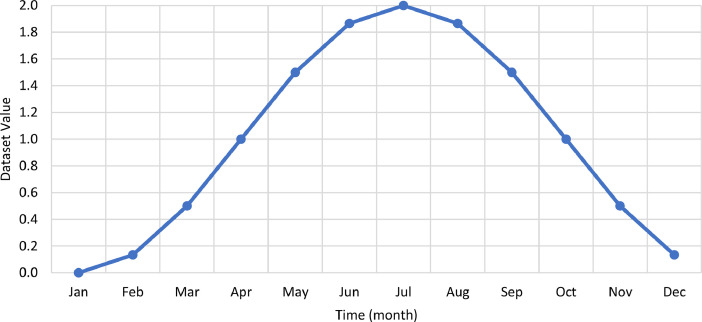
Month values used in the dataset.

The month values were represented by the trigonometric function, i.e., cosine, as displayed in Fig. 5.

**Fig. 6:**
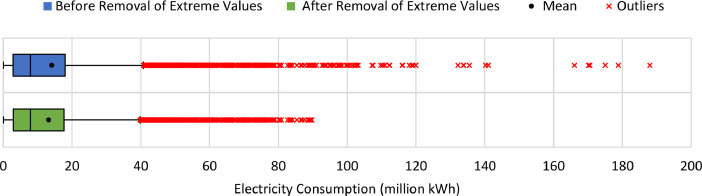
Box plots of electricity consumption pattern a) before removal of extreme values and b) after removal of extreme values.

**Fig. 7:**
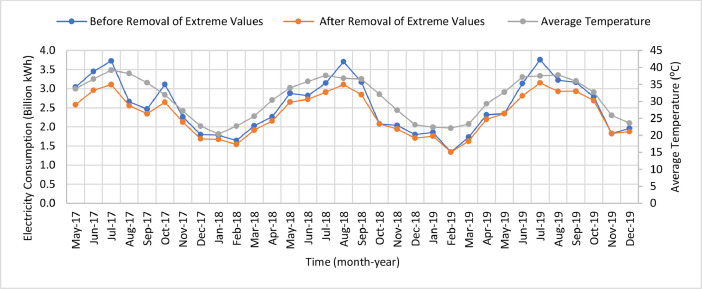
Electricity consumption pattern before and after the removal of extreme values.

**Fig. 8:**
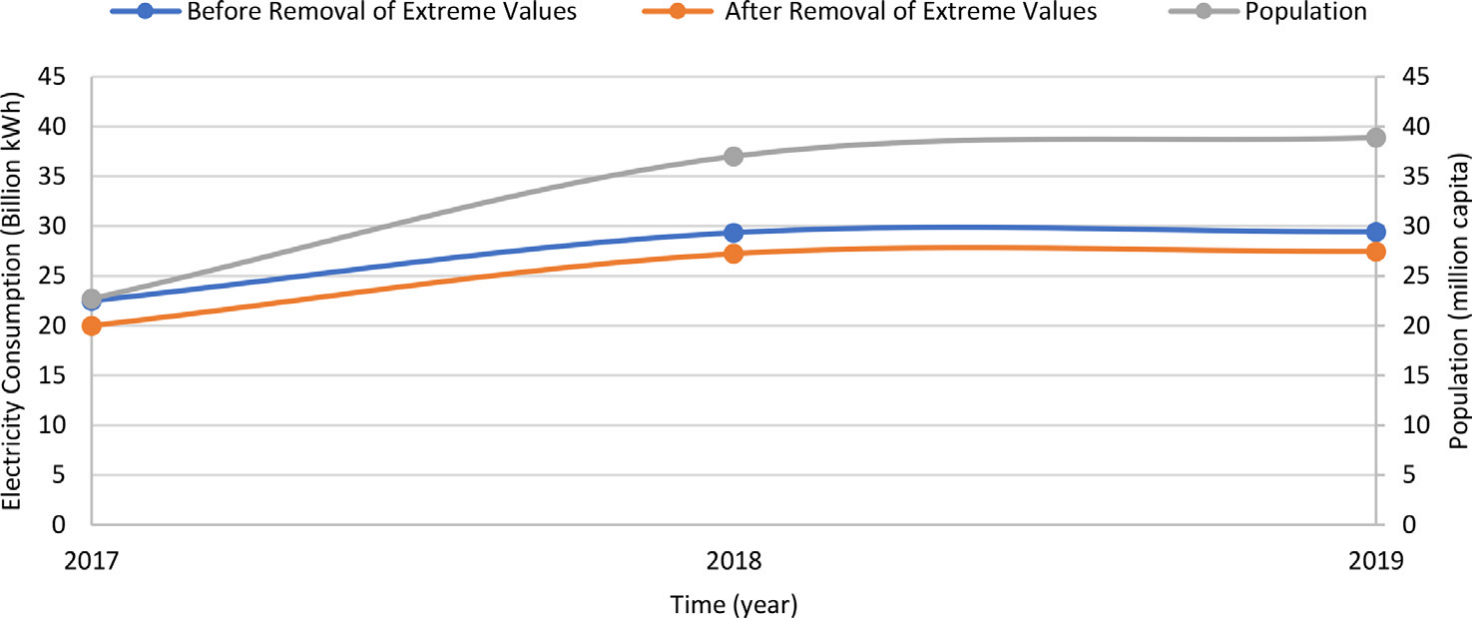
Annual electricity consumption pattern before and after the removal of extreme values.

**Fig. 9:**
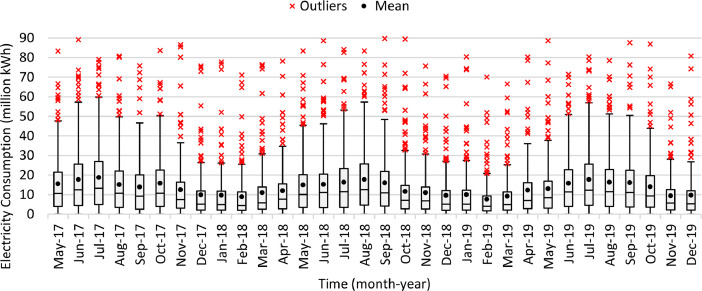
Box plots of the electricity consumption data for each month.

Table 7 illustrates the number of communities per month in the final dataset prior to modeling. Fig. 6 presents the effect of extreme value removal, whereas Fig. 7 additionally illustrates this effect with the monthly variation of temperature and Fig. 8 demonstrates this impact with the annual deviation of population. Box plots were plotted for the monthly electricity consumption as demonstrated in Fig. 9. The processed dataset was arranged in two forms to represent two datasets, namely 1) temporally ordered dataset where the electricity consumption values were ordered with respect to time and 2) randomly split dataset with a random order when trained.

**Table 7:** Description of number of communities each month post aggregation.

Time (month-year)	Number of communities	Time (month-year)	Number of communities
May 2017	166	Sep 2018	177
Jun 2017	167	Oct 2018	179
Jul 2017	166	Nov 2018	177
Aug 2017	169	Dec 2018	177
Sep 2017	169	Jan 2019	176
Oct 2017	167	Feb 2019	177
Nov 2017	170	Mar 2019	177
Dec 2017	171	Apr 2019	179
Jan 2018	172	May 2019	181
Feb 2018	173	Jun 2019	178
Mar 2018	174	Jul 2019	178
Apr 2018	179	Aug 2019	180
May 2018	178	Sep 2019	182
Jun 2018	179	Oct 2019	192
Jul 2018	178	Nov 2019	194
Aug 2018	175	Dec 2019	193
**Minimum**	**166**	**Maximum**	**194**
**Range**	**28**	**Mean**	**176**

**Fig. 10:**
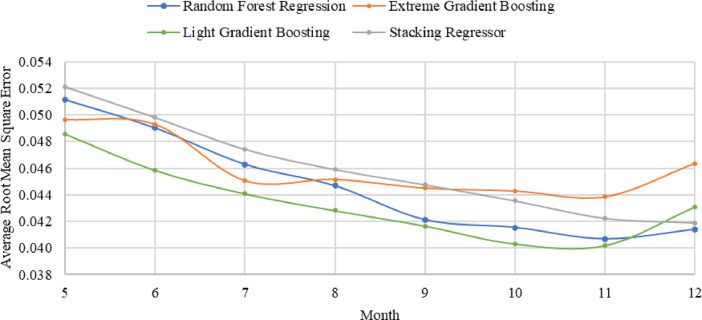
Assessment of moving cross validation method on the top four models using varying dataset size.

Table 8 and Table 9 present the evaluation results of several modes on the temporally ordered and randomly split datasets, respectively. Additionally, Table 10 presents the results of assessing different modeling techniques using moving and rolling cross validation while Fig. 10 presents the models performance through different moving cross validation with varied dataset sizes. Furthermore, Table 11 displays the best-performing models employed for investigating the feature importance in predicting electricity consumption. It compares all features to a base scenario that includes climate.

**Table 8:** Evaluation of the remaining models using different test sizes of temporally ordered dataset.

Model	Test data (%)	Root mean squared error	R^2^ (training)	R^2^ (testing)	10-fold cross validation	Mean absolute error	Median absolute error	Time taken (ms)
Artificial Neural Network	20	0.0524	0.9512	0.9049	0.9204	0.0300	0.0163	1583.6
	30	0.0479	0.9493	0.9194	0.9227	0.0287	0.0173	1434.9
	40	0.0485	0.9543	0.9095	0.9292	0.0282	0.0165	1189.3
	50	0.0519	0.9500	0.8965	0.9134	0.0301	0.0186	1065.2

Gradient Boosting Machine	20	0.0552	0.9510	0.8946	0.9093	0.0320	0.0177	1192.8
	30	0.0505	0.9517	0.9105	0.9076	0.0296	0.0174	903.6
	40	0.0474	0.9541	0.9137	0.9161	0.0282	0.0168	781.5
	50	0.0519	0.9564	0.8965	0.9229	0.0308	0.0176	655.1

Decision Tree Regression	20	0.0603	1.0000	0.8742	0.8503	0.0313	0.0135	54.0
	30	0.0652	1.0000	0.8505	0.8692	0.0332	0.0130	38.7
	40	0.0571	1.0000	0.8744	0.8757	0.0291	0.0118	42.5
	50	0.0651	1.0000	0.8369	0.8922	0.0314	0.0115	26.5

Multiple Linear Regression	20	0.0856	0.7249	0.7469	0.6888	0.0540	0.0359	3.3
	30	0.0835	0.7183	0.7551	0.6818	0.0532	0.0358	34.8
	40	0.0849	0.7273	0.7230	0.6995	0.0539	0.0349	19.1
	50	0.0846	0.7218	0.7246	0.6780	0.0533	0.0333	2.7

Lasso Regression	20	0.0904	0.7357	0.7177	0.7130	0.0557	0.0361	81.2
	30	0.0867	0.7339	0.7359	0.7125	0.0539	0.0368	53.9
	40	0.0843	0.7365	0.7266	0.7182	0.0544	0.0378	41.0
	50	0.0829	0.7331	0.7360	0.7033	0.0539	0.0371	36.3

Ridge Regression	20	0.1017	0.9138	0.6424	0.8863	0.0498	0.0287	62.1
	30	0.0887	0.9115	0.7234	0.8848	0.0442	0.0256	18.3
	40	0.0817	0.9122	0.7430	0.8650	0.0424	0.0251	17.3
	50	0.0930	0.9105	0.6679	0.8637	0.0439	0.0261	28.0

Support Vector Regression	20	0.0931	0.8099	0.7003	0.7228	0.0686	0.0595	382.9
	30	0.0974	0.8073	0.6663	0.7186	0.0686	0.0568	314.4
	40	0.1081	0.8138	0.5503	0.7473	0.0801	0.0682	247.9
	50	0.1027	0.8143	0.5948	0.7349	0.0771	0.0675	194.1

**Table 9:** Evaluation of the remaining models using different test sizes of randomly split dataset.

Model	Test data (%)	Root mean squared error	R^2^ (training)	R^2^ (testing)	10-fold Cross validation	Mean absolute error	Median absolute error	Time taken (ms)
Artificial Neural Network	20	0.0367	0.9535	0.9512	0.9292	0.0230	0.0144	1583.3
	30	0.0386	0.9499	0.9488	0.9241	0.0244	0.0150	1302.6
	40	0.0473	0.9536	0.9219	0.9224	0.0267	0.0162	1167.7
	50	0.0496	0.9579	0.9157	0.9221	0.0278	0.0159	1147.1

Gradient Boosting Machine	20	0.0417	0.9480	0.9372	0.9249	0.0257	0.0147	1027.4
	30	0.0438	0.9474	0.9339	0.9200	0.0260	0.0144	907.6
	40	0.0478	0.9512	0.9202	0.9222	0.0265	0.0148	789.1
	50	0.0474	0.9541	0.9230	0.9231	0.0272	0.0154	670.7

Decision Tree Regression	20	0.0502	1.0000	0.9088	0.8869	0.0245	0.0092	46.8
	30	0.0553	1.0000	0.8947	0.8662	0.0279	0.0109	49.1
	40	0.0644	1.0000	0.8549	0.8716	0.0296	0.0107	35.4
	50	0.0598	1.0000	0.8775	0.8703	0.0284	0.0107	29.9

Ridge Regression	20	0.0672	0.9051	0.8366	0.8889	0.0344	0.0219	33.5
	30	0.0625	0.9014	0.8656	0.7993	0.0344	0.0230	33.4
	40	0.1212	0.9100	0.4861	0.8911	0.0377	0.0228	16.9
	50	0.1072	0.9101	0.6062	0.8892	0.0375	0.0229	14.2

Support Vector Regression	20	0.0802	0.8050	0.7674	0.7697	0.0630	0.0568	389.2781
	30	0.0812	0.8002	0.7730	0.7539	0.0645	0.0579	333.4266
	40	0.0848	0.8049	0.7487	0.7569	0.0652	0.0575	271.4221
	50	0.0862	0.8040	0.7450	0.7479	0.0662	0.0603	196.3143

Lasso Regression	20	0.0832	0.7355	0.7494	0.7277	0.0542	0.0369	106.2664
	30	0.0853	0.7324	0.7498	0.7223	0.0540	0.0359	85.7459
	40	0.0864	0.7409	0.7389	0.7317	0.0534	0.0349	79.2364
	50	0.0905	0.7388	0.7190	0.7246	0.0552	0.0349	76.2522

Multiple Linear Regression	20	0.0851	0.7274	0.7376	0.7209	0.0554	0.0366	3.2779
	30	0.0869	0.7245	0.7400	0.7163	0.0554	0.0357	3.1100
	40	0.0887	0.7319	0.7245	0.7242	0.0559	0.0364	2.9420
	50	0.0909	0.7402	0.7168	0.7296	0.0565	0.0360	2.9360

**Table 10:** Assessment of the remaining models using rolling and moving cross validation methods in terms of the mean and standard deviation values of the evaluation metrics.

Models	Cross validation method	Root mean squared error	R^2^ (training)	R^2^ (testing)	10-fold cross validation	Mean absolute error	Median absolute error	Time taken (ms)
Artificial Neural Network	Moving	0.0443 ± 0.0105	0.9529 ± 0.0065	0.9190 ± 0.0407	0.9115 ± 0.0119	0.0285 ± 0.0061	0.0184 ± 0.0036	18.4 ± 3.6
	Rolling	0.0414 ± 0.0100	0.9533 ± 0.0032	0.9247 ± 0.0535	0.9192 ± 0.0098	0.0259 ± 0.0055	0.0164 ± 0.0032	1282.9 ± 526.9

Gradient Boosting Machine	Moving	0.0428 ± 0.0121	0.9675 ± 0.0035	0.9233 ± 0.0532	0.9274 ± 0.0138	0.0263 ± 0.0075	0.0155 ± 0.0042	439.9 ± 8.7
	Rolling	0.0450 ± 0.0134	0.9537 ± 0.0040	0.9101 ± 0.0674	0.9147 ± 0.0048	0.0283 ± 0.0082	0.0176 ± 0.0059	822.5 ± 231.1

Decision Tree Regression	Moving	0.0590 ± 0.0139	0.9999 ± 0	0.8538 ± 0.0797	0.8806 ± 0.0313	0.0288 ± 0.0076	0.0118 ± 0.0044	11.8 ± 4.4
	Rolling	0.0549 ± 0.0137	0.9999 ± 0	0.8662 ± 0.0933	0.8756 ± 0.0106	0.0286 ± 0.0093	0.0125 ± 0.006	36.8 ± 11.3

Multiple Linear Regression	Moving	0.0853 ± 0.0145	0.7322 ± 0.0156	0.7117 ± 0.0845	0.6962 ± 0.0155	0.0547 ± 0.0082	0.0361 ± 0.0071	36.1 ± 7.1
	Rolling	0.0846 ± 0.0119	0.7230 ± 0.0067	0.7112 ± 0.0953	0.6886 ± 0.0113	0.0539 ± 0.0064	0.0349 ± 0.0052	6.3 ± 3.4

Lasso Regression	Moving	0.0847 ± 0.0196	0.7392 ± 0.0136	0.7233 ± 0.0697	0.7181 ± 0.0142	0.0537 ± 0.0098	0.0356 ± 0.0045	35.6 ± 4.5
	Rolling	0.0835 ± 0.0162	0.7351 ± 0.0038	0.7276 ± 0.0655	0.7131 ± 0.0076	0.0535 ± 0.0072	0.0365 ± 0.0033	58.5 ± 21.8

Ridge Regression	Moving	0.0655 ± 0.0338	0.9279 ± 0.0136	0.7776 ± 0.2744	0.8804 ± 0.0318	0.0393 ± 0.0115	0.0266 ± 0.0077	26.6 ± 4.4
	Rolling	0.0618 ± 0.0277	0.9115 ± 0.0020	0.796 ± 0.3253	0.8720 ± 0.0164	0.0378 ± 0.0084	0.0253 ± 0.0035	20.9 ± 7.7

Support Vector Regression	Moving	0.0839 ± 0.0147	0.7976 ± 0.0149	0.6947 ± 0.1837	0.7159 ± 0.0294	0.0657 ± 0.0126	0.0591 ± 0.0151	59.1 ± 7.1
	Rolling	0.0843 ± 0.0148	0.8102 ± 0.0044	0.6915 ± 0.1791	0.7300 ± 0.0168	0.0664 ± 0.0127	0.0593 ± 0.0155	311.3 ± 125.4

**Table 11:** Comparison of best-performing models against base scenario (including climate) for predicting electricity consumption.

Model	Dataset order	Features included	Root mean squared error	R^2^ (training)	R^2^ (testing)	10-fold cross validation	Mean absolute error	Median absolute error	Time taken (ms)
Random Forest	Temporal	All features	0.0437	0.9915	0.9329	0.9207	0.0226	0.0096	5045.60
		Climate Features	0.1661	0.0428	0.0300	−0.0907	0.1195	0.0985	626.046

Light Gradient Boosting	Random	All features	0.0334	0.9810	0.9616	0.9451	0.0171	0.0080	698.4000
		Climate Features	0.1686	0.0482	0.0215	0.0288	0.1201	0.0922	94.9603

## 3 Experimental Design, Materials and Methods

This section describes the utilized data sources, obtained and computed external factors, preprocessing steps, applied prediction analyses, and evaluation metrics. In addition, the following research questions were addressed: 1) What is the effect of exogenous features on electricity consumption patterns in fast-growing cities?, 2) Which machine learning model obtained the best performance?, and 3) Which machine learning model demonstrated more stability and reliability?

The present study aimed to study the effect of external climatic and demographic factors on electricity consumption patterns which provided insight into the first research question. In addition, the monthly electricity consumption was forecasted on the community level in Dubai using various machine learning models that tackled the second research question. Moreover, the third research question was addressed by examining the performance and robustness of the developed models in which the size of the training dataset was varied as well as moving and rolling cross validation methods were implemented.

### 3.1 Data sources

This section demonstrates the utilized data sources, namely, Dubai Electricity and Water Authority, Dubai Statistics Center, as well as Dubai International Airport. They represent reliable governmental sources for the required datasets of electricity consumption and external factors in Dubai.

#### 3.1.1 Consumption records

Data for electricity consumption and Shams Dubai, in which household owners generate and sell solar energy through a smart power grid, were obtained during May 2017–December 2019 from an online repository, Dubai Pulse [3,4]. Table 1 shows the number of observations of the electricity consumption in each data file ranging between 1.4–1.8 million observations per file. Each file contained 8 attributes, namely billing portion, community, rate category, consumption period, calendar month, contract account, business partner, and consumption unit, which are described in Table 4. The contract account represents a unique value of a residential, commercial, industrial, or governmental site, owned by a business partner who can be associated with multiple contract accounts. In addition, an individual billing portion may contain multiple communities. Moreover, the data files also contained contract accounts enrolled in Shams Dubai initiative, in which households utilize solar panels as well as the electricity grid as an energy source. The data for these accounts contained 2 attributes called import unit and export unit instead of consumption unit as indicated in Table 2. There was no numerical relation between the electricity consumption and Shams Dubai consumption. Furthermore, rate categories indicate the type of the contract account, e.g., residential, commercial, industrial, and governmental. Rate categories as well as the number of accounts and total consumption for each rate category are shown in Table 3. Furthermore, Fig. 5 demonstrates a heat map for the electricity consumption in all communities which was generated by computing the annual average of the electricity consumption with respect to the community.

#### 3.1.2 External factors

Various external features that affected electricity consumption in the literature were determined and curated to facilitate modeling. For instance, monthly values of the ambient temperature were acquired from Dubai International Airport for the entire Emirate during the study period [5]. Fig. 3 shows the monthly variation of climate in Dubai. In addition, the annual population and number of buildings per community were obtained from Dubai Statistics Center [6]. Population data was available from 2017 to 2019, whereas the number of buildings were provided for 2019 only. Fig. 2 shows a heat map for the population of all communities in Dubai. The number of buildings data was provided for nine different types of buildings as demonstrated by Table 4 which also summarized the total number and respective percentage of each type of building. Multi-storey ratio buildings consist of two or more storeys and are built in non-conventional shapes, e.g., pyramid, cone, l-shape. Fig. 3 shows a graphical representation of the allocation of communities to sectors. The allocation of communities to sectors was implmented in this study to present a statitical summary for each sector. Table 5 summarizes the number of communities, population, and electricity consumption for each sector. Multiple features were extracted and computed from the various acquired data, such as number of sites, expatriate ratio, and building occupancy. For instance, number of sites represents the number of contract accounts with a positive consumption. Expatriate ratio was determined by dividing the number of expatriate residential accounts by total residential accounts, whereas the ratio of the population to number of buildings was utilized to obtain building occupancy. The computation of these factors was necessary to increase the accuracy of the studied models.

### 3.2 Dataset aggregation

All observations were stacked in one file then, the billing portion, business partner, and consumption period attributes were removed. Next, duplicate records were discarded to avoid any bias that may occur within the dataset. Afterwards, Shams Dubai contract accounts were excluded as there was no numerical relation between electricity consumption and Shams Dubai consumption. In addition, observations with a negative consumption were discarded to avoid the cancelation of actual instances when the data is aggregated. Additionally, observations prior to May 2017 were eliminated to limit the dataset to the study period. Moreover, all observations were aggregated with respect to the contract account. For instance, each data file contained two observations for the same contract account, where the first consumption was for a portion of the prior month, whereas the other belonged to the current month. The instances for the same month were added from two different files for the exact contract account. Instances of the same contract account in an individual file were not aggregated as they belong to two different months. Afterwards, observations with zero consumption and missing community data were discarded. The entire database containing more than 50 million observations was aggregated with respect to community and calendar month.

Multiple external factors were joined to the dataset, namely temperature, population, number of buildings, number of sites, expatriate ratio, and building occupancy. The same temperature data was utilized across all communities for each month, whereas the annual values for population and number of buildings were utilized across all months of the respective year in the dataset. Next, all instances with missing data were removed. Furthermore, various percentages of extreme values, representing outliers were discarded from the dataset to determine the optimum dataset size with the highest accuracy. For instance, the initial and final one percentile were excluded to ensure the elimination of any errors, then the consumption data was normalized [7]. Fig. 4 summarizes the data sources and the applied pre-processing methods. Next, the correlation coefficient was computed and all variables within a range of −0.2 to 0.2 were eliminated. This has been implemented to ensure the elimination of any potential sources of errors.

### 3.3 Consumption prediction

The United Arab Emirates (UAE) and other Gulf Cooperation Council (GCC) countries have reported a significant increase in energy consumption over the past decades [1]. Despite the unique consumption patterns in the region, there has been a lack of research on large-scale energy consumption at the district or city level. Therefore, this paper investigates a selection of machine learning models, some of which have not been previously deployed for consumption prediction in this region at the mentioned scale. The models explored in this study include:

• Simple regression models, such as multiple linear regression (MLR), lasso regression (LR), and ridge regression (RR), deployed as a baseline for consumption prediction.
• Support vector regression (SVR) which can model the non-linearity of the data; however, it may not perform well when there are noise in the dataset.
• Decision Tree (DT) regressor as a tree-based baseline model.
• Ensemble models, including random forest regression (RF), gradient boosting machine (GBM), extreme gradient boosting (XGB), light gradient boosting (LightGBM), and stacking regressor. Ensemble models are generally capable of efficiently modeling complex data relationships despite the correlation or linearity degree. Bagging and boosting ensemble techniques comprise many decision tree regressors as base estimators. However, bagging, as used in random forest models, has low variance, while boosting ensemble techniques, as in gradient boosting machine, extreme gradient boosting, and light gradient boosting, can reduce the prediction bias. In contrast, stack ensemble models can include baseline or complex models as base estimators, and can improve the model prediction accuracy. Nevertheless, based on the complexity of the models, they might require more computation resources and training time.
• The feedforward artificial neural network (ANN) model can extract complicated data relationships and produce high-performance models. It consists of neurons requiring some computational resources compared to baseline models.

In addition, the following evaluation metrics were utilized to assess the performance of the applied machine learning models which addresses the research questions of the present study:

(1) $$RMSE=\;\sqrt{\frac{1}{N}\sum_{i=1}^{N}{\left(X_{i}-X{}_{i}^{\prime }\right)}^{2}}$$

(2) $$R^{2}={\left(\frac{\sum_{i=1}^{N}\left(X_{i}-\bar{X_{i}}\right)\left(X{}_{i}^{\prime }-\bar{X{}_{i}^{\prime }}\right)}{\sqrt{\sum_{i=1}^{N}{\left(X_{i}-\bar{X_{i}}\right)}^{2}\sum_{i=1}^{N}{\left(X{}_{i}^{\prime }-\bar{X{}_{i}^{\prime }}\right)}^{2}}}\right)}^{2}$$

(3) $$MAE=\;\frac{1}{N}\sum_{i=1}^{N}\left|X_{i}-X{}_{i}^{\prime }\right|$$

(4) $$MedAE=median\;\left(\left|X_{1}-{X^{\prime }}_{1}\right|,\;\ldots ,\;\left|X_{N}-X{}_{N}^{\prime }\right|\right)$$

(5) $$CV_{k}=\;\frac{1}{k}\sum_{i=1}^{k}R_{i}^{2}$$

where N is the data size, $X_{i}$ and $X{{}^{\prime }}_{i}$ are the observed and forecasted values, respectively, and $\bar{X_{i}}$ and $\bar{X{{}^{\prime }}_{i}}$ are the observed and forecasted means, respectively. In addition, varying percentages of both datasets were utilized to test the load forecasting models.

Tables 8 and 9 show the performance of ANN, GBM, DT, MLR, LR, RR, and SVR, whereas the behavior of the remaining models, namely RF, XGB, LightGBM, and staking regressor, was presented by Abdallah et al. [1]. Moreover, two CV schemes, i.e., moving, and rolling, were utilized to evaluate the ability of the models to train new data accurately. In moving CV, the models were tested using varying dataset sizes to define the optimum length which yields an accurate estimation. Next, the models were trained using the optimum dataset length while sliding by an individual month every prediction instant. On the other hand, rolling CV implemented a 12-month long dataset to forecast the first instance, then the dataset length was increased by an individual month every prediction instant. A detailed elaboration of the utilized machine learning models as well as moving and rolling cross validation methods was provided by Abdallah et al. which was followed in the present study [1]. Fig. 10 illustrates the average RMSE obtained by RF, stacking regressor, XGB and LightGBM applying moving CV scheme when tested on various dataset lengths. Moreover, the average and standard deviation of the forecasting accuracy for the remaining models implementing rolling and moving CV procedures are demonstrated in Table 10. The feature importance was investigated by utilizing the best performing models to predict the electricity consumption with all features versus a base scenario consisting of climate in Table 11.

## Limitations

During the data collection, a few limitations were observed which may impact the interpretation of the presented data. The obtained climatic features were collected from a single location, i.e., Dubai International Airport, which may not fully represent the various geographical locations of different communities in Dubai. In addition, the population records were obtained for each community on an annual basis; hence, the same value was utilized across all months of the year for each community.

## Ethics Statement

The authors have read and follow the ethical requirements for publication in Data in Brief and confirming that the current work does not involve human subjects, animal experiments, or any data collected from social media platforms.

## CRediT authorship contribution statement

**Mariam Hosny:** Formal analysis, Visualization, Writing – original draft. **Omnia Abu Waraga:** Investigation, Validation. **Manar Abu Talib:** Methodology, Writing – review & editing. **Mohamed Abdallah:** Project administration, Conceptualization, Supervision.

## Data Availability

Monthly Electricity Consumption with Exogenous Factors Datasets (Reference data) (Mendeley Data).
